# Why Did Home Care Personal Support Service Volumes Drop During the COVID-19 Pandemic? The Contributions of Client Choice and Personal Support Worker Availability

**DOI:** 10.1177/11786329231210692

**Published:** 2023-11-14

**Authors:** Emily C King, Katherine AP Zagrodney, Prakathesh Rabeenthira, Travis A Van Belle, Sandra M McKay

**Affiliations:** 1 VHA Home HealthCare, Toronto, ON, Canada; 2 Dalla Lana School of Public Health, University of Toronto, Toronto, ON, Canada; 3 Institute of Health Policy Management & Evaluation, University of Toronto, Toronto, ON, Canada; 4 Canadian Health Workforce Network, University of Ottawa, Ottawa, ON, Canada; 5 Public Health Agency of Canada, Toronto, ON, Canada; 6 Department of Physical Therapy, University of Toronto, Toronto, ON, Canada; 7 Ted Rogers School of Management, Toronto Metropolitan University, Toronto, ON, Canada

**Keywords:** Home care services, Personal Support Worker, COVID-19, service holds, visit cancellations, missed care, workplace absences

## Abstract

Home care personal support service delivery decreased during the COVID-19 pandemic, and qualitative studies have suggested many potential contributors to these reductions. This paper provides insight into the source (client or provider) of reductions in home care service volumes early in the pandemic through analysis of a retrospective administrative dataset from a large provider organization. The percentage of authorized services not delivered was 17.2% in Wave 1, 12.6% in Wave 2 and 10.5% in Wave 3, nearing the pre-pandemic baseline of 8.9%. The dominant contribution to reduced home care service volumes was client-initiated holds and cancellations, collectively accounting for 99.3% of the service volume; missed care visits by the provider accounted for 0.7%. Worker availability also declined due to long-term absences (which increased 5-fold early in Wave 1 and remained 4× above baseline in Waves 2 and 3); short-term absences rose sharply for 6 early-pandemic weeks, then dropped below the pre-pandemic baseline. These data reveal that service volume reductions were primarily driven by client-initiated holds and cancellations; despite unprecedented decreases in Personal Support Worker availability, missed care did not increase, indicating that the decrease in demand was more substantial and occurred earlier than the decrease in worker availability.

## Introduction

Home care services are a vital support used by 6% of the Canadian population to enable them to continue to live in their homes and communities while receiving care.^1^ Personal Support services are the most commonly delivered form of care, representing approximately 80% of all formal home care delivery.^2,3^ It has been well-documented that, as in other countries,^4,5^ home care Personal Support service delivery volumes in Ontario, Canada decreased during the first wave of the COVID-19 pandemic^3,6^; decreasing by 18.9% at the population-level in April 2020 and rebounding to 94% of pre-pandemic levels by September 2020.^3^

While Sinn et al’s population-level data from Ontario Health’s Home Care Dataset provides an accurate overview of service volumes, it does not provide insight into whether these service reductions were due to client choices to decline home care (eg, due to fear of infection, public health guidelines or changes to the availability of family caregivers) or due to decreased availability of home care Personal Support services (eg, due to worker shortages). Conversely, qualitative studies that have explored client and family concerns about continuing to receive home care during the pandemic^5,7-9^ and home care provider’s choices regarding their engagement with work during this time^10-12^ are not able to speak to the degree of impact of each of these factors on overall changes to home care service delivery volumes.

The purpose of this paper is to bridge this gap by providing insight into the source (client or provider) of reductions in Personal Support service provision through the first 3 waves of the COVID-19 pandemic.

## Methods

### Design

This retrospective cohort study leveraged longitudinal administrative data from a large home care organization that provides Personal Support services in the Greater Toronto Area of Ontario, Canada to provide an overview of service utilization by clients and the availability of workers to provide this service. The open cohort comprised all clients who were provided with publicly-funded Personal Support services by this organization from August 26, 2019 until June 27, 2021, and all Personal Support Workers involved in providing this care. Approval for this study was provided by the University of Toronto Research Ethics Board (REB# 40086).

### Data

Client-level data describing service utilization included the number of Personal Support visits provided each week, the start and end dates of any client-initiated service holds, and the dates of any cancelled visits and missed care. Service holds are defined as a client-initiated suspension of home care Personal Support services, whether due to choice or hospitalization. A cancelled visit is recorded when a client chooses to cancel a single scheduled home care visit. In contrast, a missed care event occurs when the organization does not deliver an individual scheduled visit (eg, if a PSW calls in sick and no replacement worker can be found). Client demographics (age, sex and acuity level) were used to describe the sample. Client acuity was defined as low, medium or high based on ‘emergency response level’ (urgency of service) scores received as part of the home care service referral package. These scores are determined by the referring Home and Community Care Support Services organization and are designed to support prioritization of care and wellness calls in emergencies.

PSW-level data included both demographics (age and sex) and descriptors of workforce participation during the study period, including workforce entry and exit, short-term absences (sick days, planned days off and other unplanned absences) and longer-term leaves of absence.

### Analysis strategy

During each week of the sample, the total authorized service volume (the amount of care allocated by the funder – the Home and Community Care Support Services) is described in terms of numbers of visits provided and not provided (due to holds, cancellations or missed care). The volume of service not received during each week of the study is estimated for each client on hold using their average weekly service volumes in the 4 weeks prior to placing a hold. The total volume of non-received service each week is estimated as the sum of non-receipt due to holds, cancelled visits and missed care and is presented as a percentage of the total authorized service volume.

The findings are presented as descriptive summaries (means, ranges) and through the presentation of time series plots to summarize changes in service levels and PSW availability.

## Results

The cohort includes a total of 14 735 clients who were served by 2007 Personal Support Workers. The average client age was 76.9 years (range: 1.3-106.9 years), 65.8% were female. 38.2% of clients were deemed to have ‘high acuity’ (at greatest risk if Personal Support is not provided), 28.9% to have medium acuity and 27.8% to have low acuity; acuity scores for the remaining 5.1% of clients were not available. The PSWs averaged 45 years of age (range: 18-77 years) and 96.0% were female.

### Reductions in service volumes: Holds, cancellations and missed care

**Figure 1.**
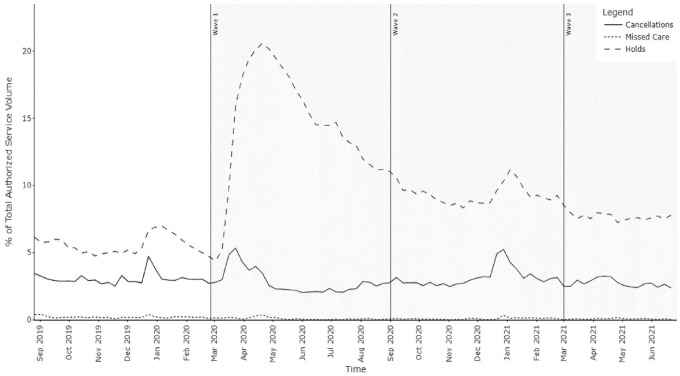
Contribution of service holds, cancellations and missed care to overall service volumes.

Visit volumes decreased throughout Waves 1 to 3, compared to the 6 months prior to the pandemic (Figure 1). For home care clients, non-receipt of care can be due to: (1) client-initiated holds (ie, non-receipt of care for an extended period, whether due to choice or hospitalization), (2) clients cancelling individual visits and (3) ‘missed care’, which is a scheduled visit for which the organization does not provide service. Overall, the average percentage of authorized service not delivered in Wave 1 of the pandemic was 17.2%, dropping to 12.6% in Wave 2 and 10.5% in Wave 3, nearing the pre-pandemic baseline of 8.9%. In our sample, non-receipt of service peaked in mid-April 2020, when 24.5% of authorized visits were not delivered. As shown in Figure 1, the dominant contribution to the overall reduction in home care service volumes was client-initiated holds, followed by client-initiated cancellations, collectively accounting for 99.3% of reduction in service volume during the pandemic portions of the study period. Notably, missed care by the provider accounted for only 0.7% of the total reduction in service volume (0.09% of the total authorized service volume) from the start of Wave 1 until the end of the study.

**Figure 2.**
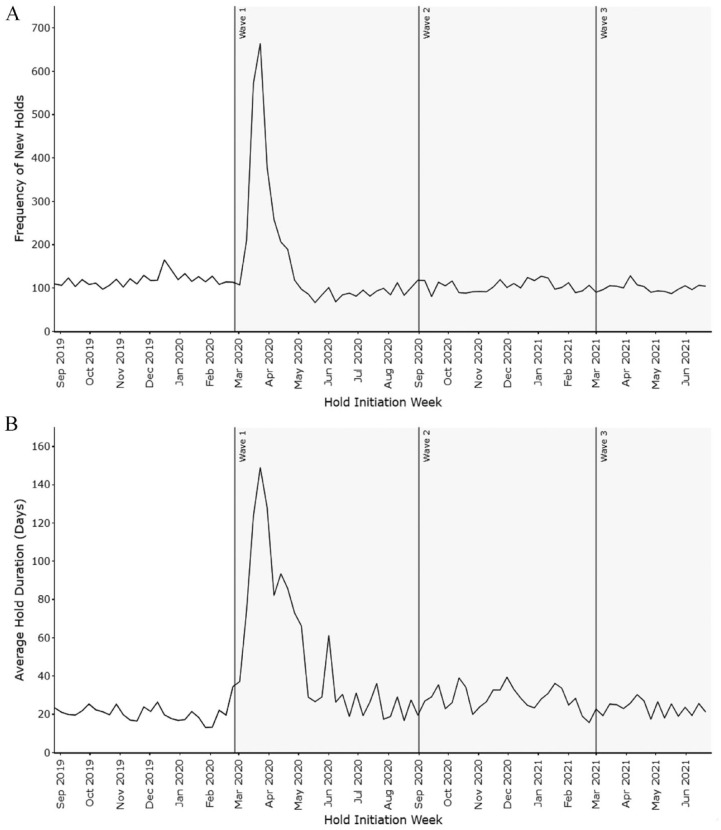
Characteristics of new client-initiated service holds. (A) shows the number of holds initiated each week, while (B) shows the average hold duration by week of hold initiation.

Client-initiated service holds were the most substantial contributor to the service volume reductions observed during the pandemic periods. As a percentage of the total reduction in service delivery, client-initiated holds accounted for 83.0% of this reduction during Wave 1, 74.3% in Wave 2 and 73.4% in Wave 3 until Jun 27, 2021. Compared to typical pre-pandemic hold rates that accounted for 5.6% of all authorized service, hold levels rose sharply near the beginning of Wave 1 and remained elevated throughout Waves 2 and 3 (Figure 1). This effect was most pronounced during the first wave of the pandemic, when the rate of new holds peaked at 5.6 times the pre-pandemic baseline. After the first 9 weeks, the rate of new holds approached the pre-pandemic baseline level (Figure 2A), after which new holds became less frequent, dropping below baseline. However, many clients, especially those who placed holds during the initial 10 weeks of Wave 1, remained on hold for extended periods, peaking at an average hold duration of approximately 5 months for those who initiated holds in the first weeks of the pandemic (Figure 2B). The increase in new service holds in Wave 1 was primarily driven by dramatically increased hold rates amongst medium- and low-acuity clients. Although clients with high acuity typically have the highest rates of home care service holds (eg, due to hospitalization), service hold rates for high acuity clients increased by only 18.4% in Wave 1, compared to a 55.8% increase for medium-acuity clients and 78.1% for low-acuity clients. Notably, the rate of holds due to hospitalization did not increase across this time period.

Client-initiated visit cancellations were the second most substantial contributor to reduced Personal Support visit volumes. Pre-pandemic, an average of 3.1% of visits were cancelled each week (Figure 1). With the exception of an increase in cancellation rates during an 8-week period early in Wave 1 and the usual seasonal increase over the winter holiday period, cancellation rates remained at or below baseline levels throughout the pandemic portions of the study period. Looking specifically at the contribution of cancellations to service authorized but not received during each wave, client-initiated cancellations accounted for 16.5% of the services not received during Wave 1, 24.9% in Wave 2 and 25.9% in Wave 3.

Missed care by the organization was the final contributor to lower service volumes, accounting for 0.6% of the overall reduction in Personal Support visit volumes in Wave 1, and 0.7% in each Waves 2 and 3 (Figure 1). In Wave 1, rates of missed care peaked 4 weeks after the peaks in client-initiated service disruptions (holds and cancellations), but rapidly returned to lower levels than the pre-pandemic baseline.

### Reductions in PSW availability: Leaves and short-term absences

**Figure 3.**
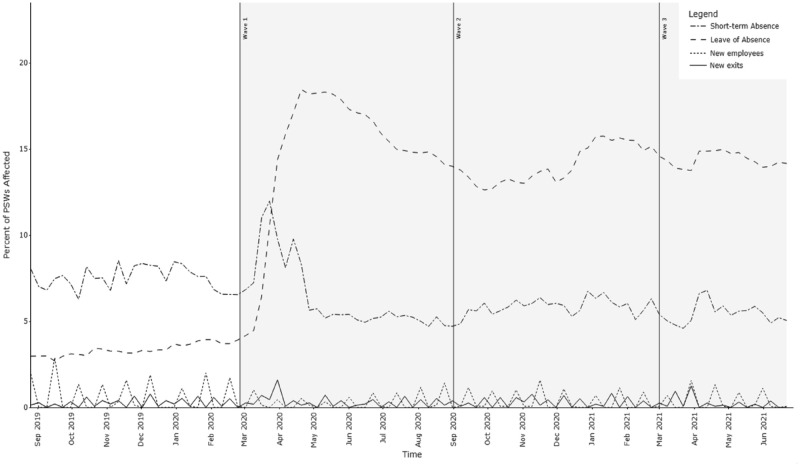
The contributions of Leaves of Absence, Short-Term Absences and workforce entry and exit to reductions in PSW availability.

Health system capacity to deliver home care personal support service is typically tied closely to the availability of Personal Support Workers. PSW availability did drop sharply early in Wave 1, but, proportionally, the decline in PSW availability (Figure 3) was lower than the contemporaneous reduction in demand for home care service. Both extended leaves of absence and short-term absences contributed to reduced availability of PSWs, with leaves of absence representing the much larger contributor to reduced provider availability. Leaves of absence rose sharply early in Wave 1 to over 5 times the pre-pandemic baseline (peaking at 18.4%, compared to a pre-pandemic baseline of 3.3%) and reduced slightly in Waves 2 and 3, but remained 4 times higher than typical pre-pandemic levels (average 14.2%, range: 12.6%-15.7%). In March and April of 2020, the top reported reasons for new leaves of absence were childcare (due to the closure of schools and daycares or online schooling requiring parental support^13^), health-related concerns (though not COVID-19 infection) and a provincial ‘single-site’ policy which prevented PSWs who worked in institutional long-term care from also working in home care.^14^ In Wave 2, the proportion of PSWs self-isolating following COVID-19 exposure was also a substantial contributor to leaves of absence. Short-term absences (due to illness, personal emergencies or other unplanned absences) also rose sharply in the first 6 weeks of the pandemic, but then dropped below pre-pandemic baseline rates.

## Discussion

Recent publications have highlighted reductions in home care service volumes in Ontario^3,6^ and internationally^4,5^ during the COVID-19 pandemic, but have been unable to provide clarity around the reasons for these reductions. Speculation around reasons for these decreases has pointed to 2 factors identified in the qualitative literature: client choice not to receive service and a shortage of home care workers to provide necessary care (eg, Jones et al) (2021). This home care organization-level data, which reflects similar drops in service volumes to the population-level report provided by Sinn et al, reveals that volume reductions during the first 3 waves of the pandemic were driven heavily by clients’ choices not to receive service, expressed primarily through placing service holds. This decision was made less frequently by clients and families with the most acute service needs, in alignment with the population-level finding that Ontarian home care clients with dementia (who have greater average service needs) reduced their service levels at lower rates than the general home care population (16% vs 19%).^3,6^ While home care provider availability is more typically the primary driver of home care service delivery volumes, the reductions in provider availability remained lower than reductions in demand for service through these first 3 waves of the pandemic. This is reflected in the low, stable rates of missed care throughout the study period.

While the present dataset does not directly address reasons for client choices to decline home care during the COVID-19 pandemic, extensive qualitative literature has identified numerous factors that contributed to these decisions. Fear of infection and a desire to adhere closely to public health advice to reduce contacts where possible have been identified frequently as drivers of these decisions, particularly during the first wave when little was known about the transmission, severity and treatment of COVID-19, and when personal protective equipment was in short supply throughout the world.^5,7,9^ In this context, many clients and families decided that the safest option for them was to place their home care services on hold, or to only allow a limited group of familiar home care workers to provide visits and cancel visits for which alternative care providers were scheduled.^5,7-9^ For many families, these decisions to forego publicly-funded care came at significant personal cost to the family caregivers and were not sustainable in the long term.^5,9,15-18^ For others, fundamental changes in the work arrangements of family caregivers (eg, due to furloughs or work-from-home arrangements) may have increased the feasibility of reducing their reliance on paid care.^19,20^

In contrast with US and Dutch studies,^4,5^ restrictions on worker availability were not a notable driver of reduced service volumes in the present study. While it was less substantial and occurred more slowly than the decline in demand for care, there were notable decreases in the availability of Personal Support Workers, driven primarily by leaves of absence. While the majority of PSWs remained willing to work despite anxiety about contracting and transmitting COVID-19,^12,21^ the finding that leaves of absence for PSWs increased 5-fold during Wave 1, affecting nearly one-fifth of this workforce, echoes other literature about the early phases of the pandemic. These leaves were largely driven by lack of childcare (due to school and daycare closures), caregiving responsibilities and fear (either their own or fears expressed by family members) of infection and transmission to vulnerable loved ones.^10-12^ Interestingly, while the rate of short-term absences did rise sharply in the first weeks of the pandemic, it dropped below the pre-pandemic baseline level in mid-April, 2020 and remained below pre-pandemic levels throughout the remainder of the study period. This may have reflected a combination of factors including feeling a duty to work^12,22^; restricted travel, appointment and social opportunities related to public health restrictions^13^; broad efficacy of public health precautions^23^; and potentially a ‘healthy worker’ effect as some workers with pre-existing conditions took leaves of absence to reduce their risk of exposure to COVID-19.

## Limitations

This study used administrative data from a single home care organization and is therefore reflective of how clients and workers experienced this organization’s specific policies, practices and workplace protections. The administrative dataset was captured for service planning rather than research purposes, incorporates inputs from multiple sources (clients, the home care organization and the quasi-governmental Home and Community Care Support Services) and is not always complete. While the structured data format supports consistency and quantitative analysis, it provides limited insights regarding factors that influenced clients’ decision making regarding their choices to receive or decline service and Personal Support Workers’ decision-making regarding their choices to continue to work or to go on a leave of absence. Additional research will be required to better understand these choices.

## Conclusions

Home care Personal Support service volumes were much lower in the early phases of the pandemic (Waves 1-3) than before March 2020. Examination of organization-level data reveals that this was primarily driven by clients’ choices to place service holds and cancell service, which aligned with public health guidelines to reduce their contacts and potential exposures. Although there were unprecedented decreases in Personal Support Worker availability, missed care did not increase. This indicates that while both supply of and demand for care decreased in Wave 1, the decrease in demand was more substantial and occurred in advance of the decrease in worker availability. This was fortunate, but by no means inevitable. Further investigation of the factors driving clients’ decisions to place service holds or cancel visits, and contributing to PSWs’ leaves and short-term absences is required to understand how to better support these individuals, inform planning and ensure consistent home care service availability in future crises.
